# New findings of silica nanoparticles induced ER autophagy in human colon cancer cell

**DOI:** 10.1038/srep42591

**Published:** 2017-02-14

**Authors:** Fujing Wei, Yimin Wang, Zewei Luo, Yu Li, Yixiang Duan

**Affiliations:** 1 Research Center of Analytical Instrumentation, Key Laboratory of Bio-resource and Eco-environment, Ministry of Education, College of Life Sciences, Sichuan University, Chengdu 610065, PR China

## Abstract

Nanoparticle-induced autophagy has been extensively studied, however, real time information about the endoplasmic reticulum involved autophagic process (ER autophagy) induced by nanomaterials remains unknown. In this work, silica nanoparticles (SNPs) were synthesized with characteristics of low toxicity, good biocompatibility and excellent water dispersibility to treat cells. Results show that either low concentration (10 μg/mL) or high concentration (200 μg/mL) of SNPs could increase the quantity of processing from microtubule-associated protein 1-light chain 3-I (LC3-I) to the other variant of LC3 (LC3-II). Interestingly, the level of autophagy induced by the SNPs is associated with the treated time but not the concentrations of SNPs. Importantly, for the first time, SNP accumulation in ER was discovered through co-localization analysis, which incurs ER autophagy. These new findings about SNPs-induced ER autophagy could open an effective way for securely designing silica-based nanoparticles and enable us to know more about ER autophagy.

A lot of research work has demonstrated that amorphous silica nanoparticles (SNPs) are relatively biocompatible^1^, and the SNPs have been widely used in gene transfection, drug delivery, biosensing and bioimaging due to their unique properties of tunable pore size, high surface area, and ease of modification^2^,^3^,^4^,^5^. In addition, a great deal of attention has been paid to the nanotoxicity of SNPs, which is suggested depend on their specific surface area and sizes^1^,^6^,^7^. Previous reports have shown that nanoparticles can be internalized by cells through multiple protein dependent endocytosis and micropinocytosis^8^,^9^. Taken all together, the endocytosis mainly rely on clathrin or clathrin independent pathway^8^,^10^,^11^. Lim *et al*.^12^,^13^ have proposed that clathrin-mediated endocytosis is regulated by coating proteins with the involvement of membrane invagination (~200 nm). The endosome related to internalization of plasma membrane has a massive amount of membrane proteins and receptors on the surface. As for non clathrin-mediated types, raft-mediated caveolae/lipid involves internalization (~60–80 nm), while macropinocytosis/phagocytosis involves invaginations (>0.2 μm)^13^,^14^.

The internalized SNPs have been regarded as a kind of novel autophagy activators in cellular physiological activity^15^. However, cell autophagy, a process of the degradation of intracellular materials, is a conserved catabolic process from yeast to mammals^16^. It is induced by various stimuli, such as deprivation of nutrient, aggregated proteins and unwanted organelles other than SNPs^11^. Usually, these stimuli result in the double-membrane-bound autophagosomes (APs) to engulf cytoplasmic constituents which include agminated abnormal protein and damaged organelles and so on. Then the APs are fused with the lysosomes to form autolysosomes (ALs) to degrade these constituents, and over 32 autophagy-related genes (Atgs) regulate this process^17^. In the regulation mechanisms, cell autophagy acting as a housekeeper balances the biological functions of organelles or proteins, prevents the excessive materials from making an adverse effect on cells, and maintains the synthesis, degradation and subsequently recycling of cellular components^18^. Therefore, cell autophagy is always associated with aging of the organism, cancer, neurodegenerative diseases, infection diseases, and so on. Generally, autophagy can be classified into two types: non-selective autophagy and selective autophagy. The former is induced by starvation, and degrades cytoplasm^19^,^20^, while the latter needs particular receptor to target specific materials, such as mitophagy for clearance of damaged mitochondria, aggrephagy for degradation of aggregating proteins, lysophagy for target of damaged lysosomes^19^.

In the past few years, many research groups have proposed that ER, an integral and elaborate membrane organelle for folding and modifying secretory proteins, can itself be captured by autophagy if it is damaged^21^. This process is called “ER autophagy”^22^. ER autophagy is one of the selective autopahgy, and its role always links to unfolded protein response (UPR) that aims to maintain cell homeostatic control^23^. Researchers have affirmed that ER stress mediates numerous responses during brain ischemia^24^,^25^. The accumulation of aggregated proteins or aberrant proteins always links to neurodegenerative diseases, such as Parkinson, Alzheimer and prion-related diseases^26^,^27^. Therefore, ER autophagy plays an important role in these diseases, and Mochida *et al*.^28^ have showed that ER autophagy, under stress conditions, is required for ER turnover. To date, although SNPs induced apoptosis and necrosis have been reported^29^,^30^, it is still necessary to find more sufficient evidence to elucidate some phenomenon in ER autophagy, especially considering the fact that there is no report with regard to the ER autophagy induced by SNPs. The newly discovered receptors for ER autophagy, including Atg 39 that localizes to the perinulcear ER and incurs autophagic segregation of part of the nucleus and Atg 40 that is enriched in the cortical and cytoplasmic ER and delivers ER subdomains into APs in yeast, and FAM134B in mammalian, have been reviewed in a paper published in *Nature*^31^. Khmints *et al*.^32^ have showed that ER autophagy is mediated by FAM134B, and suggested that FAM134B helps to fragment the ER into APs. Thus there will be a good reason for further exploration of the SNPs-induced ER autophagy.

To this end, SNPs were synthesized in this work to treat human colon cancer cells (HCT-116) for investigating SNPs-induced autophagy. SNPs decorated with fluorescein isothiocyanate (FITC) were used to track the accumulation of SNPs in cells. Microtule-associated protein1 light chain 3 (LC3), a key protein involved in “cargo” delivery and development of the double membrane, have two forms of LC3, called LC3-I and LC3-II, involved in auophagosome formation^33^. The amount of LC3-II is related to the extent of autophagosome formation, thus LC3-II acts as a special protein associated with autophagosome membranes^33^. Another protein, p62, which is involved in cargoes including misfolded, aggregated proteins and dysfunctional organelles delivered to the APs, was also used in this work. p62, as a signaling adaptor, bind ubiquitinated proteins and LC3-II and then finally results in targeting of substrates to the APs^34^,^35^. Importantly, in this work, the co-localization assays provided some evidence to illustrate the accumulation SNPs in ER, resulting in the ER autophagy.

## Results

### Synthesis and characterization of SNPs

**Figure 1:**
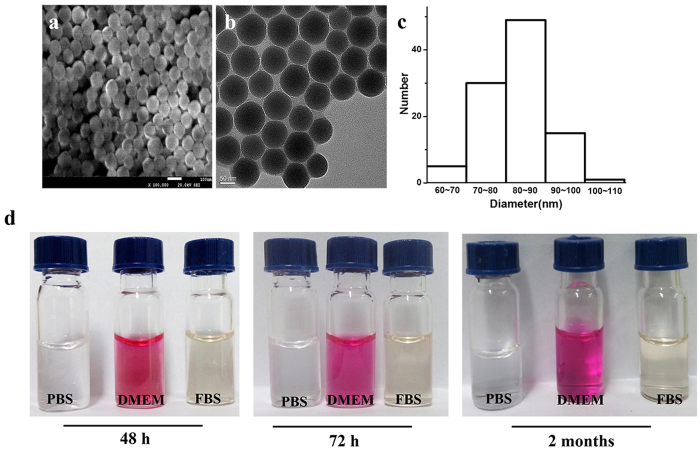
Characterization of SNPs. The sizes of SNPs were evaluated by SEM (**a**) and TEM (**b**). The SNPs exhibit a near-spherical shape with good dispersibility. (**c**) The size distribution of SNPs by SEM. The average diameter of the SNPs is roughly 86 nm. (**d**) The stability of SNPs dissolved in PBS, DMEM, and FBS after 48 h, 72 h, and 2 months, respectively.

The SNPs were synthesized by referring to the Stober methods as described in Materials and Methods sections^36^,^37^. Transmission electron microscopy (TEM), scanning electron microscopy (SEM) and dynamic light scattering (DLS) were used to evaluate the characteristics of SNPs. As shown in Fig. 1a,b, near-spherical SNPs with uniform distribution are formed showing favorable monodispersity. The size distribution of SNPs with a mean diameter of roughly 86 nm viewed from SEM by software Gatan DigitalMicrograph is shown in Fig. 1c. To evaluate the stability of SNPs, in subsequent experiments, SNPs were dissolved in PBS, FBS and DMEM and stored at 4 °C for 48 h, 72 h and two months, respectively. All groups of solutions were clear and no difference from initial state was observed. Additional experiments were conducted with a stable monodisperse suspension of SNPs obtained after DLS assays. Results also demonstrate that the SNPs have a good stability in Supplementary Figure 1.

### Synthesis and characterization of FITC-SNPs

**Figure 2:**
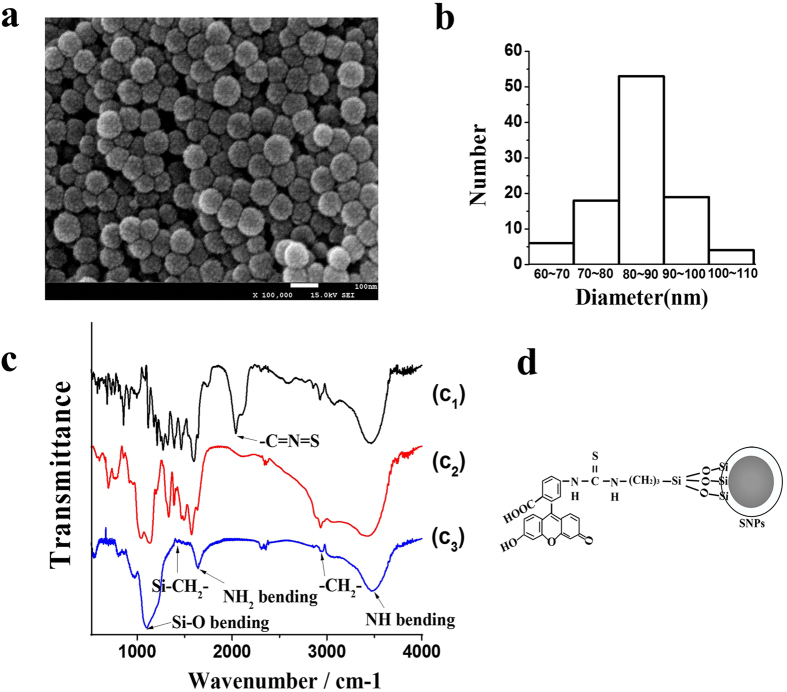
Characterization of SNPs with SEM, and FTIR. The SEM images (**a**) of FITC-SNPs showing an average diameter of approximately 86 nm (**b**). (**c**) FTIR spectra of the FITC (c_1_), FITC-APTES (c_2_) and FITC-SNPs (c_3_) to identify SNPs successfully modified by FITC. (**d**) The mechanism of FITC-APTES decorating SNPs, where the FITC-SNPs were washed until the supernatant fluorescence fell into a low intensity, as shown in Supplementary Figure 2.

In order to trace the locations of SNPs in HCT-116 cells, the SNPs were modified with FITC. The detail of the modification process is described in the Method section. The ultrastructural FITC-SNPs was exemplified by SEM (Fig. 2a) with the mean size of roughly 86 nm (Fig. 2b). Fourier transform infrared spectrometer (FTIR) was used to identify the chemical functional groups of FITC-SNPs surface (Fig. 2c) and the mechanism of the FITC-modified SNPs was studied and is shown in Fig. 2d. In Fig. 2c, the vanished peak at 2,061 cm^−1^ substantiate that the FTIC was triumphantly united with APTES, which is attributed to isothiocyanato group (-C=N=S) as a connection between FITC and APTES would change once FITC is completely conjugated with APTES. The strong peak at 1,100 cm^−1^ in Fig. 2c is assigned to Si-O stretching vibration of the SiO_2_ matrix^38^, and the weak peak at 1,424 cm^−1^ is attributed to the Si-CH_2_ bending of -Si-(CH_2_)_2_-NH_2_- of APTES. The small peak at 2,960 cm^−1^ is assigned to stretching vibration of methylene (-CH_2_-) group incurred from the hydrolysis and subsequent condensation of -Si-(CH_2_)_2_-NH_2_ of APTES. Functionalization of the SNPs with FITC-APTES was confirmed by the peak at 1,642 cm^−1^ corresponding to the characteristic bending of -NH_2_ and another asymmetric peak at 3,486 cm^−1^ attributed to –N-H stretching.

### Cytotoxicity of SNPs on HCT-116 cells

**Figure 3:**
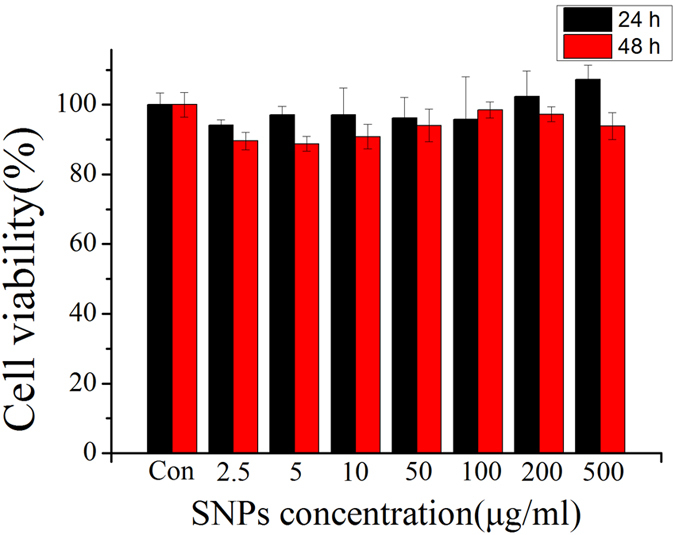
The viability of the HCT-116 cells affected by SNPs. The viability of HCT-116 cells exposed to SNPs with different concentrations ranging from 2.5 μg/mL to 500 μg/mL for 24 and 48 h, respectively.

Conventionally, there are many methods, such as MTT, XTT and LDH release assays, to test the cell toxicity of nanoparticles. Here, the cell viability was evaluated by CCK-8 after the HCT-116 cells were exposed to different concentrations of SNPs (2.5, 5, 10, 50, 100, 200 and 500 μg/mL) for 24 and 48 h, respectively. Fig. 3 shows that comparing with the control group, the SNPs barely reduced the cell viability even at high concentration of SNPs (500 μg/mL). Overall, the SNPs did not remarkably induce cell death and show no significant toxicity to HCT-116 cells. The results adequately indicate that the synthesized SNPs are low toxicity.

### SNPs sucked by HCT-116 cells and accumulated in ER

**Figure 4:**
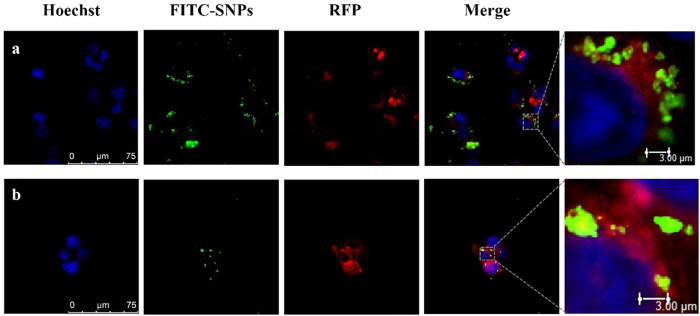
SNPs accumulation in ER and lysosomes. HCT-116 cells were firstly transfected with ER-RFP and Lyso-RFP, respectively, and then FITC-SNPs were used to treat the transfected HCT-116 cells. The confocal imaging shows (**a**) FITC-SNPs co-localization of Lyso-FRP and (**b**) co-localization with ER-RFP.

In order to further examine the process of SNPs in cells, the uptake process of SNPs in HCT-116 cells was examined. Lyso-RFP and ER-RFP were used to label lysosomes and ER, respectively. The green fluorescence of FITC-SNPs located in HCT-116 cells as shown in Fig. 4 indicates that SNPs can be taken up by HCT-116 cells and even arrived at nucleus. In addition, one can also see that the green fluorescence partly overlay with red fluorescence. These results indicate that FITC-SNPs were not only co-localized with lysosomes but with ER. Therefore, FITC-SNPs were taken up by HCT-116 cells and then accumulated in both ER and lysosomes.

### Cell autophagy stimulated by SNPs

**Figure 5:**
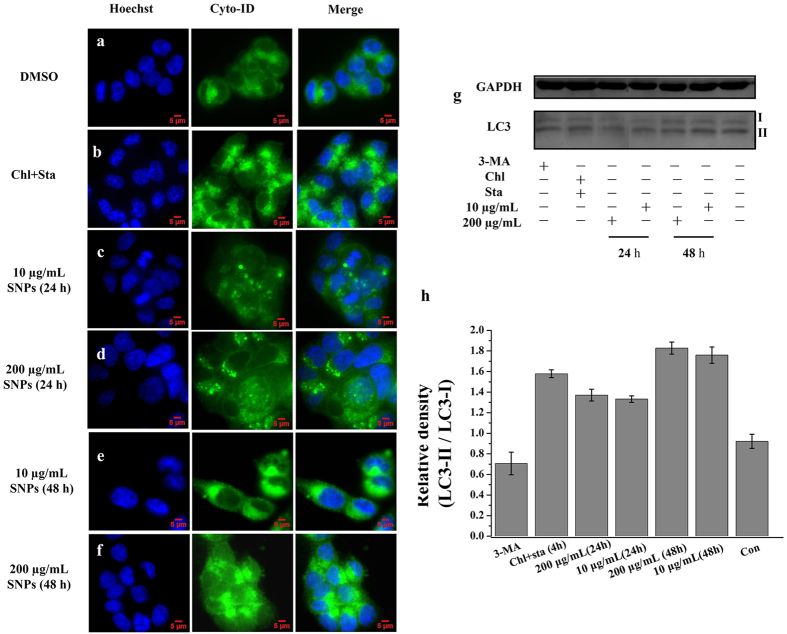
Effect of SNPs on the activation of autophagy. The merge of the Cyto-ID fluorescence dye and Hoechst 3342 in HCT-116 cells treated with DMSO (**a**), 12.5 μM chloroquine coupled with starvation for 4 h (**b**), 10 μg/mL SNPs for 24 h (**c**), 200 μg/mL SNPs for 24 h (**d**), 10 μg/mL SNPs for 48 h (**e**), or 200 μg/mL SNPs for 48 h (**f**). The result of western blot of various treatments on HCT-116 cells (**g**), GAPDH was used as a loading control, and the image was analyzed by software of AzureSpot (**h**). More results referring full length of blotting bands were shown in Supplementary Figure 4. These blotting bands were obtained from the same experimental conditions.

SNPs may put stress on the HCT-116 cells and subsequently induce autophagy responses. In this work, according to the manual, Cyto-ID function was employed to testify whether autophagy was activated by SNPs in HCT-116 cells through labelling autophagy compartments. Cyto-ID fluorescent images show visible green particles not only in the cells treated with chloroquine but also those cells treated with various concentrations of SNPs for different time as illustrated in Fig. 5. Cells treated for 48 h obviously displayed increased level of autophagy than those treated for 24 h, while there was no significant distinction when the concentrations of SNPs were changed from 10 to 200 μg/mL under the same treatment time. In addition, flow cytometry was also used to measure the level of autophagy resulted from SNPs, and the results (supplementary Figure 3) also testify that SNPs induced cell autophagy rather than inhibit autophagy flux.

To further understand cell autophagy and explore whether SNPs indeed affect autophagy via simply targeting APs or inducing the stress of cell organelles, western blot assays were utilized. Previous studies have demonstrated that the level of LC3-II is closely associated with autophagic activity^39^.

Thus urea-SDS-PAGE was utilized in western blot assays to well separate LC3-I and LC3-II^39^. The results of western blot in Fig. 5g,h show that SNPs stimulated cell autophagy, and increased the expressing level of LC3-II. Cells treated with 48 h obviously display increased level of autophagy than that of cells treated for 24 h, while there was non-significant distinction when the concentrations of SNPs were changed from 10 to 200 μg/mL under the same treatment time. Thus these results are in good agreement with Cyto-ID test.

### SNPs induce cell autophagy

**Figure 6:**
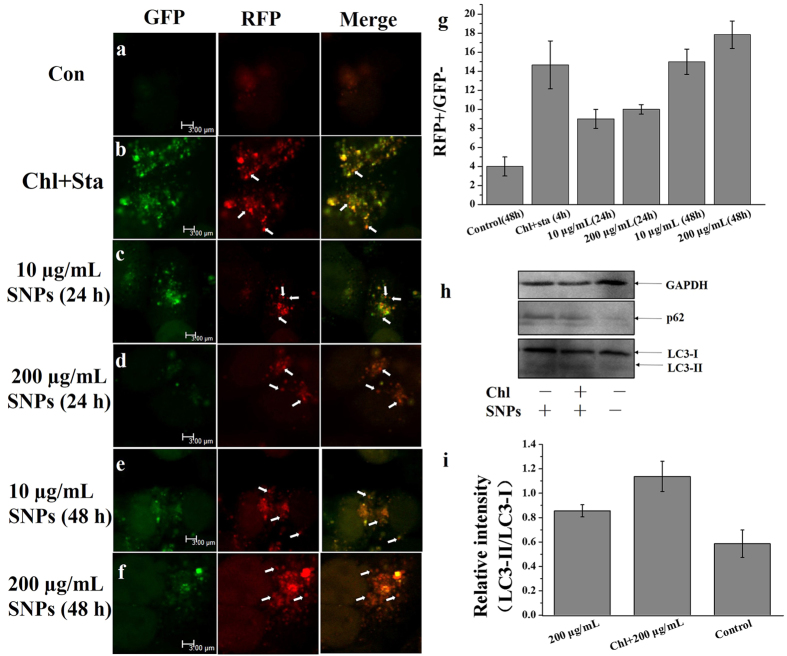
SNPs induced autophagy. Control without any treatment **(a)**, positive control, with 12.5 μM chloroquine and starvation for 4 h (**b**), HCT-116 cells were treated with 10 μg/mL SNPs for 24 h (**c**), 200 μg/mL for 24 h (**d**), 10 μg/mL SNPs for 48 h (**e**), 200 μg/mL SNPs for 48 h (**f**), GFP-RFP-LC3 results analyzed by LCSM (**g**), and all the group under the same regulation conditions. The result of western blot of HCT-116 cells treated with 200 μg/mL SNPs for 48 h or 12.5 μM chloroquine for 1 h, then treated with 200 μg/mL SNPs for 48 h (**h**). Image of western blot analyzed by software of AzureSpot (**i**). More results referring full length of blotting bands were shown in Supplementary Figure 5. These blotting bands were obtained under the same experimental conditions.

In order to further testify that SNPs induce cell autophagy rather than inhibit the autophagy flux, in our work, HCT-116 cells were stably transefected with a marker gene, tandem fluorescent-tagged LC3 (GFP-RFP-LC3) lentiviral vector as the operation manual to monitor the LC3 tranlocation. Results in Fig. 6a–f also show that SNPs stimulated the autophagy flux and Fig. 6g indicates that the red fluorescence increased with longer treatment time, which suggests that SNPs induce the increasing number of APs, and thus the autophagy in HCT-116 cells was induced by SNPs instead of inhibiting autophagy flux. Figure 6h,i show that the level of LC3 was increased upon SNPs treatment for longer time, and the result was also testified by p62. In addition, chloroquine increase the level of LC3–II, compared with cells treated with 200 μg/mL SNPs for 48 h, which further indicates that SNPs induce the cell autophagy instead of inhibiting the late stage of autophagy.

### The effects of SNPs on ER autophagy

**Figure 7:**
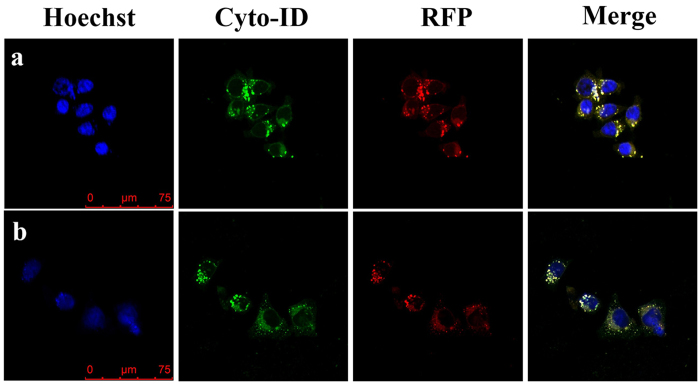
SNPs induce the ER autophagy. (**a**) The co-location of HCT-116 cells between Lyso-RFP (red) and Cto-ID (green); (**b**) ER-RFP co-localized with Cyto-ID. The RFP was used to label ER and lysosomes. The Cyto-ID was used to label autophagic vacuoles.

Cyto-ID and western blot have well demonstrated that the SNPs incur the cell autophagy. Considering the location of SNPs in HCT-116 cells, it was expected that the accumulated SNPs may also incur ER autophagy. To this end, Cyto-ID was used to monitor autophagic vacuoles and RFP was used to label ER and lysosomes, respectively. Figure 7a shows that most of the APs were located in lysosomes, while Fig. 7b shows that the APs were also co-located with ER. In addition, results in Fig. 4 have shown that there was a high accumulation level of SNPs in ER. Thus we can say that the confocal image (Fig. 7) shows that the co-localization of autophagic vacuoles, ER and lysosomes is the consequence of the SNPs-induced ER autophagy.

## Discussion

In this work, we have identified that the synthesized SNPs display special structure and physicochemical properties. CCK-8 experiments have affirmed the SNPs at the concentrations ranging from 0.5 μg/mL to 500 μg/mL, is almost no toxicity to HCT-116 cells. Many experiments in this work show that SNPs can induce the cell autophagy, and even show that SNPs can induce ER autophagy in HCT-116 cells.

With the treatment of different concentrations of SNPs, the fluorescence intensity of Cyto-ID indicates that the level of autophagy is associated with the treated time but not the concentration of SNPs. The results of Fig. 5c–f show that higher concentrations of SNPs did not result in obvious increase of fluorescence intensity at the same treatment time. On the other hand, the longer treated time obviously increase the cell autophagy at the same concentration. Western blot also shows the same conclusion. The level of LC3-II/LC3-I does not obviously increase, compared 10 μg/mL and 200 μg/mL SNPs for 24 h, or the contrast of 10 μg/mL and 200 μg/mL for 48 h. However, the longer treatment time can increase the level of LC3-II, compared 10 μg/mL for 24 h and 10 μg/mL for 48 h or contrast 200 μg/mL for 24 h and 200 μg/mL for 48 h. Overall, compared with control group, it was easy to identify that the longer treated time increased the level of cell autophagy in HCT-116 cells, but the various concentrations of SNPs are not closely associated with the level of cell autophagy.

The tandem fluorescent of GFP-RFP-LC3 results as characterized by various levels of yellow and red dots directly show the occurrence of the process of APs to APL, indicating that increasing the treatment time at the same concentration of SNPs can activate the autophagy flux, and thus incur the accumulation of APL shown as red dots.

Thus we tentatively concluded that prolonging the treated time can increase the level of autophagy, whereas the effect of the concentration of SNPs is negligible in the process. All of those results suggest that SNPs are capable of inducing autophagy and made an effect on ER and then incurred ER autophagy in HCT-116 cells. Schütz, I. *et al*.^40^ had reported that internalized SNPs can accumulate in lysosomes, resulting in lysosomal dysfunction in Hela cells. Similarly, SNPs accumulating in ER as shown in Fig. 4 may indicate that SNPs have an effect on ER through an unknown mechanism. Furthermore, ER is closely connected with cell autophagy. There is a consensus that autophagosomal membranes are mainly originated from ER^41^, and some APs can be directly obtained from ER subdomains^42^,^43^,^44^,^45^,^46^. In our work, the co-localization of Cyto-ID and RFP (ER-RFP and Lyso-RFP) (Fig. 7) collectively show that SNPs induce the cell autophagy, especially for ER autophagy. It is expected that the merged yellow fluorescence by green fluorescence and red fluorescence in Fig. 7 indicate the ER autophagy induced by SNPs. The mechanism of the SNPs-induced ER autophagy and the internalization mode of cells are not clear at this point, and further investigation is needed to better interpret the process.

**Figure 8:**
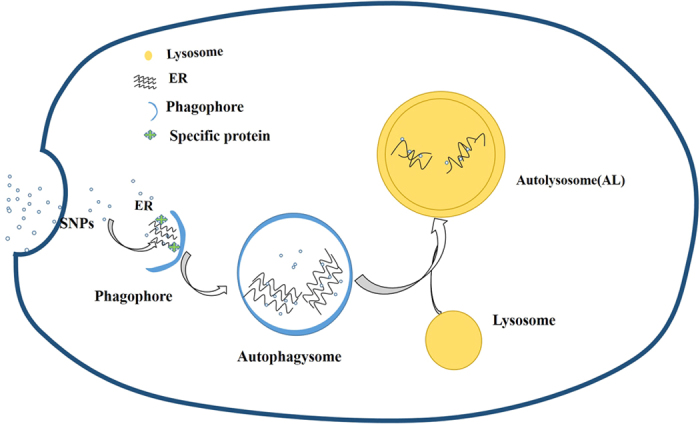
Diagram of ER autophagy in HCt-116 cells induced by SNPs.

Overall, as shown in Fig. 8, SNPs were taken up by HCT-116 cells and then accumulated in ER eventually incurring the ER autophagy. Therefore, our study for the first time proposed that SNPs-induced ER autophagy in HCT-116 cells serves as a response of the SNPs-interfering in ER in order to maintain ER normal function. Undoubtedly, our finding is beneficial for designing silica-based nanoparticles, further exploration of ER autophagy, enable us to know more about ER autophagy and provide insight into selective autophagy in pathological associated with ER stress.

## Materials and Methods

### Materials

Ultrapure water of 18.2 MΩ cm (Ulupure, Chengdu, China. Tetraethyl orthosilicate (TEOS) was purchased from Kelong, China. Aminopropyltrimethoxysilane (APTES) was purchased from Sigma-Aldrilch, mainland China. Ammonia solution (25%) was obtained from Guangdong Guanghua Sci-Tech Co. Ltd, China. LC3 primary and LC3 secondary antibodies were received from Cell Signaling Technology, USA. GAPDH antibody was purchased from Zen BioScience, China. Bicin chonininc acid (BCA), SDS-PAGE Kit and the RealBand 3-color Regular Range Protein Marker were obtained from Beijing Solarbio Science & Technology, China. Dulbecco’s Modified Eagle Medium (DMEM) and FBS were purchased from GiBco, USA. Phophate-buffered saline (PBS) was purchased from Biosharp, USA, and then dissolved into deionized purified water. Radio Immunoprecipitation Assay (RIPA) and Cell Counting Kit (CCK-8) were obtained from Vazyme biotech co. ltd, Nanjing, China. Cyto-ID was purchased from Enzo life sciences, Switzerland. Hoechst was purchased from sigma, Lysosomes-RFP, ER-RFP were obtained from Life Technologies, USA. Tandem fluorescent-tagged LC3 (GFP-RFP-LC3) lentiviral vector was obtained from Gene Chem, Shanghai.

### Synthesis of silica nanoparticle

The SNPs were prepared utilizing previously modified Stober methods^36^,^37^. Briefly, before adding 100 μL of TEOS, the ethanol solution was mixed with 2 mL ammonia and 4 mL water and then the mixture was kept stirring at room temperature for 12 h. The SNPs were separated by centrifugation (12,000 r/min) for 15 minutes and then washed for three times with ultrapure water after rinsing three times with ethanol, and finally, dispersed in PBS for cell culture or in ethanol for subsequent surface-modification. The SNPs were performed by transmission electron microscopy (TEM, Tecnai G2 F20 S-TWIN, USA), and scanning electron microscopy (SEM, JSM-7500 F JEOL, Japan). The size of SNPs in SEM was analyzed by Gatan DigitalMicrograph software.

### Synthesis of FITC-labelled silica nanoparticle

The FITC-labelled SNPs were obtained from Zhou *et al*.^46^. Simply, 2 mg FITC and 200 μL APTES were mixed in 10 mL ethanol solution with stirring overnight to obtain FITC-modified APTES. Then 10 mL SNPs were dispersed in ethanol and mixed with FITC-APTES. The mixture was stirred at 50 °C for 6 h before keeping at room temperature for 2 h, and then FITC-labelled SNPs were collected after washing three times with water and dialyzing at least for 48 h in dark condition until there is no fluorescence in supernatant. Eventually, FITC-SNPs were characterized by FTIR (PerkinElmer, USA) and SEM. The size of FITC-SNPs viewed from SEM was analyzed by Gatan DigitalMicrograph software.

### Biological stability

SNPs were shaken by ultrasound for 10 min. Then SNPs were added into PBS, DMEM and FBS respectively, at the same conditions at 4 °C. The pictures were taken in 48 h, 72 h and two months. And the hydrodynamic diameter of the SNPs was determined by dynamic light scattering (DLS, Malvern, UK).

### Cell viability assays

HCT-116 cells (5 × 10^3^ cells per well) in logarithmic phase were incubated in 96-well bottomed-flat plate at 37 °C with 5% CO_2_. After 24 h, cells were treated with a series of dilutions of SNPs 0–500 μg/ml for 24 h or 48 h, respectively. Cells without any treatment were set as control groups. Then, cell viability was evaluated by using CCK-8 test. The plates were incubated at 37 °C for 4 h. The absorbance of cells treated with SNPs was measured by an automatic microplate reader (iMark, BIO-RAD, USA) at 450 nm, 10% CCK-8 solution was added in each well. The results were assessed as an average of three identical measurements.

### SNPs localization in HCT-116 cells

The uptake of SNPs by HCT-116 cells was visualized through confocal microscopy. The cell organelles were labeled with special fluorescent probe. First, cells were incubated overnight with lysosome-RFP, in which RFP was fused with lysosomal-associated membrane protein 1 (Lamp1) to indicate the localizations of lysosomes, or transfected with ER-RFP, where RFP was fused with ER signal sequence of calreticulin and KDEL. The transfected cells were treated with SNPs for 24 h, then fixed with 4% paraformaldehyde and staining nucleus with Hoechst 33342. The fluorescent images of the cells were obtained by laser scanning confocal microscope (LSCM, TCS SPS II, Leica, Germany).

### The fluorescence of Cyto-ID

To measure if autophagy in HCT-116 cells were resulted from the SNPs, Cyto-ID, an autophagy detection kit, was used to selectively label autophagic vacuoles and monitor autophagy in live cells^47^. Briefly, about 1 × 10^5^ cells per well were seeded into a 24-well plate in DMEM with 10% FBS, and the cells were incubated at 37 °C for 18–24 h until they reached a confluent of 60–70%. Cells were treated with 50 μM 3-MA, or 12.5 μM chloroquine combined with starvation for 4 h, 10 μg/mL, and 200 μg/mL SNPs for 24 h and 48 h, respectively. Cyto-ID green detection reagent, consisting of 1 μL Hoechst 33342, 100 μL Cyto-ID reagent per 1 mL cell culture medium supplemented with 10% FBS was used to stained cells. The plate was stored in dark for 30 min at 37 °C. After washing with PBS supplemented with 5% FBS for three times to remove excess fluorescent dye, the cells were fixed with 4% paraformaldehyde for 20 min and rinsed for three times. Then the coverslip containing cells was placed on a microscope slide. Finally, a standard FITC filter and DAPI filter of wide-field fluorescence microscopy (IX83, Olympus, Japan) were used for imaging the autophagic vesicles and nucleus signal respectively.

### Flow cytometry

HCT-116 cells (2–3 × 10^5^ cells/well) were incubated in 6-well plate at 37 °C in 5% CO_2_. When the confluence of cells reached ~80%, they were conducted for the next steps of the experiment, where various stimuli to induce or inhibit autophagy. Then 0.35 ml of freshly diluted Cyto-ID Green Detection Reagent (1 ml indicated free culture medium containing 5% FBS or 1 ml 1X Assay Buffer, containing 1 ul Cyto-ID solution) were added into the plate. And then the plates were incubated for 30 min at 37 °C in dark. Then cells were washed and were collected and centrifuged (2,000 rpm/min, 5 min) at the end of the treatment with trypsin and resuspended in 0.5 ml of 1 × Assay Buffer. The samples were transfered into 5 ml nonsterile FACS tubes and immediately analyze by flow cytometry (BD FACSCalibur^TM^ Flow Cytometer).

### Western blot assays

As a standard method to measure autophagy, western blot technique has been wildly used. Here, western blot was used to evaluate the levels of LC3-I, LC3-II, and GAPDH. Cells were incubated with 10 μg/mL or 200 μg/mL SNPs for 24 h and 48 h, respectively. 12.5 μM chloroquine coupled with starvation for 4 h was utilized as positive control, but 50 μM 3-MA treated cells and none treatment cells as negative controls. Then HCT-116 cells were lysed on ice with RIPA. The concentrations of protein of HCT-116 cells were measured by BCA. Lysates were loaded on to Urea-SDS-PAGE^39^,^48^. Briefly, 0.9 g urea was added into 15% separating gel (5 mL in total) and 0.36 g Urea was added into 5% condensing gel (2 mL in total). The Urea-SDS-PAGE was loaded with samples and electrophoresed at 80 V for 30 min and at 120 V for 60 min then transferring at 200 mA for 60 min. The membranes were blocked with sealing fluid before incubating primary antibodies at 4 °C overnight. Second antibodies connected horseradish peroxidase were incubated for 1 h at room temperature. The concentrations of primary and secondary antibodies of LC3 were used at dilution ratio of 1:2,000, primary and secondary antibodies of GAPDH were 1:10,000. After rinsing with TBST for three times, samples were incubated by enhanced chemiluminescence reagent that can specifically recognize secondary antibodies by using chemiluminescence (Azure C300, USA), then the image was analyzed through the software of Azure C300 (AzureSpot).

### Gene transfection with viral vectors

HCT-116 Cells (5 × 10^4^ cells per 6-well) were stably transfected with a marker gene, tandem fluorescent-tagged LC3 (GFP-RFP-LC3) lentiviral vector as the operational manual to monitor the LC3 translocation. Briefly, HCT-116 cells were incubated overnight to reach approximately 70% confluence. Then fresh medium was employed to replace the culture medium, and transfection was conducted by a multiplicity of infection of 100, thus resulting in an infection rate of more than 80%. After 12 h, the culture medium was replaced with fresh medium, and the cells were incubated for additional 96 h for subsequent experiments.

### SNPs induced autophagy

The transfected HCT-116 cells were used to assess the autophagy flux induced by SNPs. Briefly, about 1 × 10^5^ transfected cells were cultured overnight on the 24-well plate and then treated with Cyto-ID assay as described above. After the cells were fixed on microscope slides, the cells were observed under a LSCM. Under the condition of the acidic environment of the lysosome, GFP was fused to LC3 rapidly loses fluorescence, while RFP retains fluorescence in the lysosome^49^,^50^. Therefore, the autophagic flux resulted by SNPs was measured by counting the red puncta that indicates intact auophagic flux, and the cell with yellow puncta resulting in mixing of red puncta and green puncta that indicates autophagylysosome.

### SNPs induced ER autophagy

Firstly, HCT-116 cells were transfected with RFP to label ER or lysosomes at 37 °C over 16 h. After the transfected cells being washed by PBS supplemented with 5% FBS, the cells were treated with the SNPs and kept incubating at 37 °C. Cells were washed for three times and then incubated with Cyto-ID reagent to label autophagic vacuoles at 37 °C for 30 min. Finally, the cells were rinsed and viewed through LSCM.

### **S**tatistical analysis

The experiments as mentioned above all were conducted at least three times. The size of SNPs or FITC-SNPs in SEM was assessed by 100 nanoparticles by Gatan DigitalMicrograph. Cyto-ID results were analyzed by Olympus CellSens software. Western blot results were analyzed by AzureSpot software. Co-localization results were analyzed by LAS AF Lite software. Flow cytometry results were analyzed by FlowJo.

## Additional Information

**How to cite this article**: Wei, F. *et al*. New findings of silica nanoparticles induced ER autophagy in human colon cancer cell. *Sci. Rep.* **7**, 42591; doi: 10.1038/srep42591 (2017).

**Publisher's note:** Springer Nature remains neutral with regard to jurisdictional claims in published maps and institutional affiliations.

## Supplementary Material

Supplementary Information
