# Supplementary material for: New findings of silica nanoparticles induced ER autophagy in human colon cancer cell
Source: Sci Rep. 2017 Feb 14;7:42591. doi: 10.1038/srep42591 (PMC5307363; doi:10.1038/srep42591)
Supplement: Supplementary Information [file srep42591-s1.pdf]

New findings of silica nanoparticle induced ER autophagy in human colon cancer cell

Fujing Wei [a, 1], Yimin Wang [a, 1], Zewei Luo [a], Yu Li [a], Yixiang Duan [a,*]

[a] Research Center of Analytical Instrumentation, Key Laboratory of Bio-resource and Eco-environment, Ministry of Education, College of Life Sciences, Sichuan University, Chengdu 610065, PR China. [1] These authors contributed equally to this work. * Correspondence and requests for materials should be addressed to Yixiang Duan (E-mail address: yduan@scu.edu.cn).

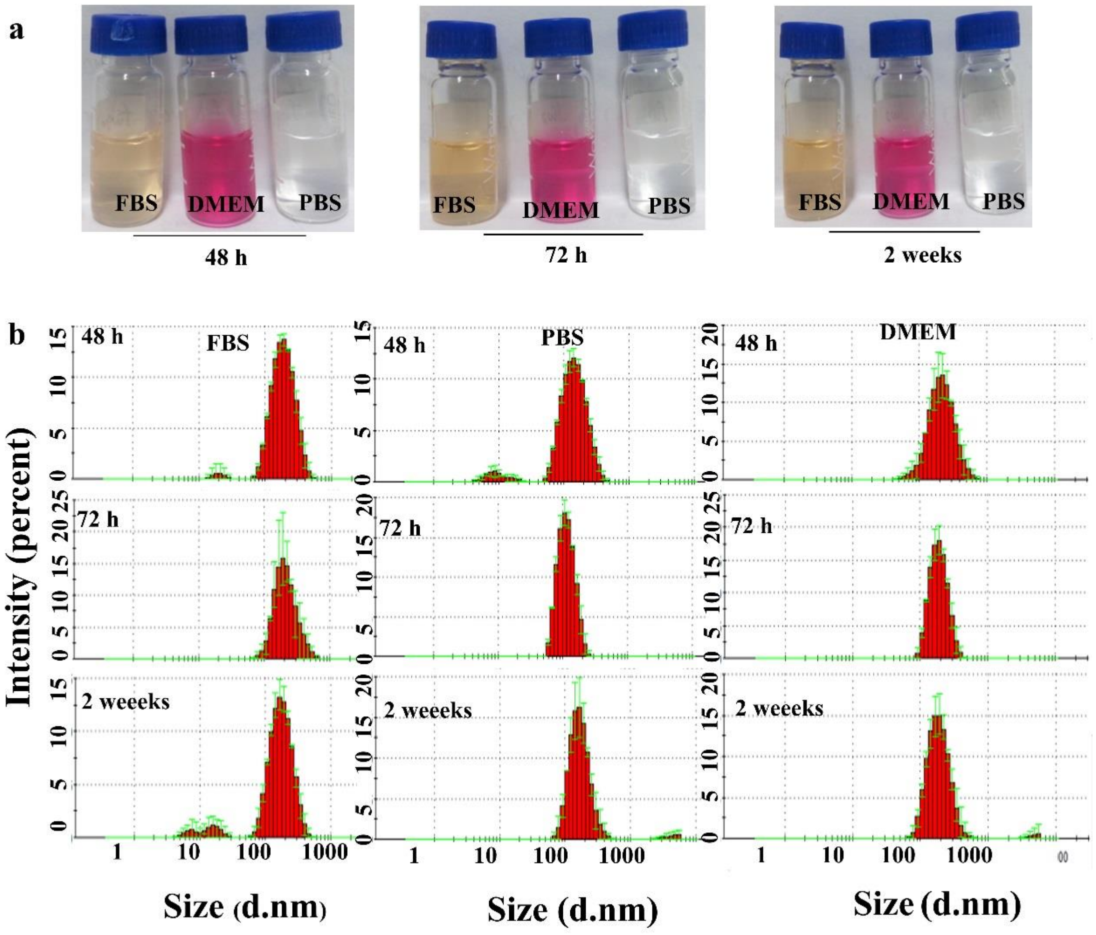

Supplementary Figure 1. Characterization of SNPs. (a) The stability of SNPs dissolved in FBS, DMEM, and PBS after 48 h, 72 h, and 2 weeks, respectively. (b)

DLS was used to evaluate the stability of SNPs in FBS, DMEM, and PBS at 48 h, 72 h, and 2 weeks, respectively.

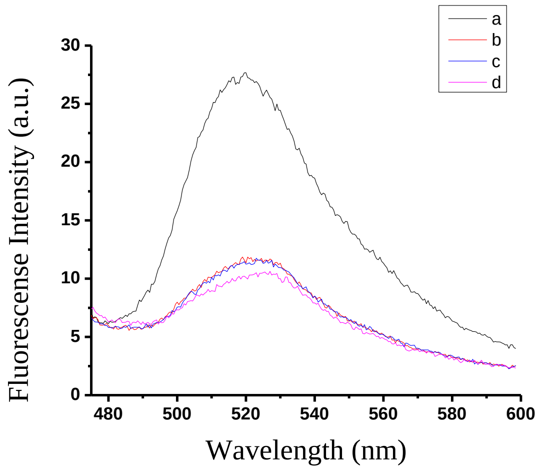

Supplementary Figure 2. The supernatant fluorescence spectra of SNPs. The supernatant fluorescence gets weaker and weaker with the washing times increasing from 1 to 4 (a-d).

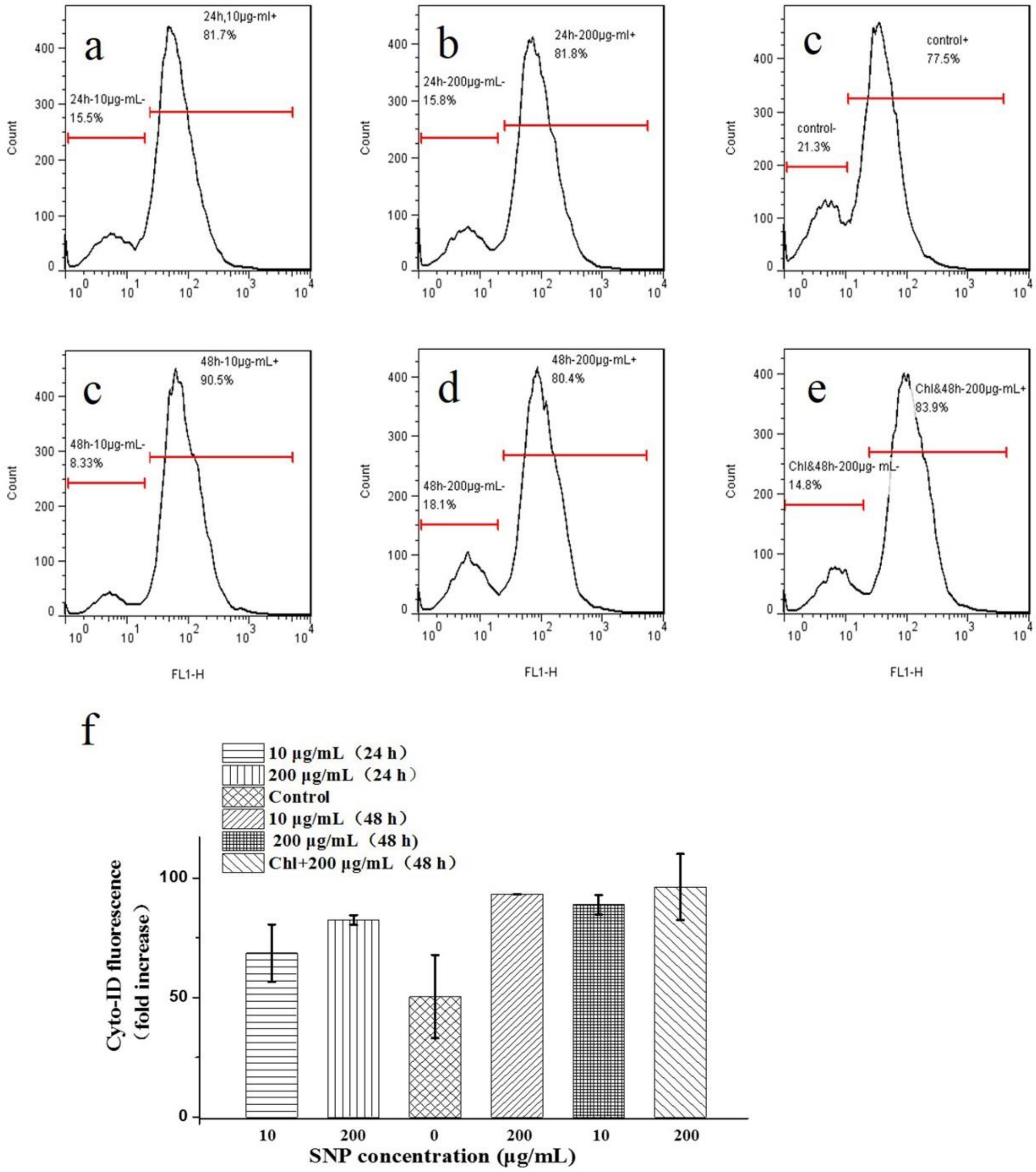

Supplementary Figure 3. Flow cytometry evaluated autopahgy induced by SNPs. HCT-116 cells were treated with 10 μg/mL SNPs for 24h (a), 200 μg/mL for 24h (b), as a control, without any treatment (c), 10 μg/mL SNPs for 48h (d), 200 μg/mL SNPs for 48h (e), 12.5 μM Chloroquine for 1 h, then treated with 200 μg/mL SNPs for 48h (f). Flow cytometry results were analyzed by FlowJo (g), and all the groups were performed under the same condition.

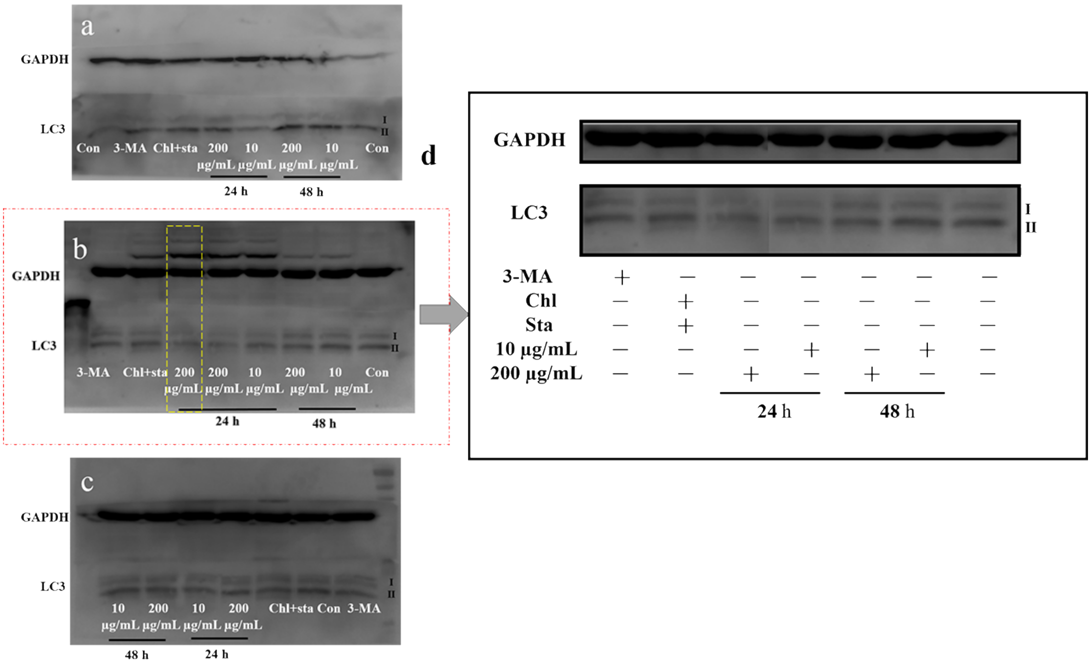

Supplementary Figure 4. SNPs increase the expression level of LC3. All the blotting bands were obtained from the same experimental condition. d is cropped from b.

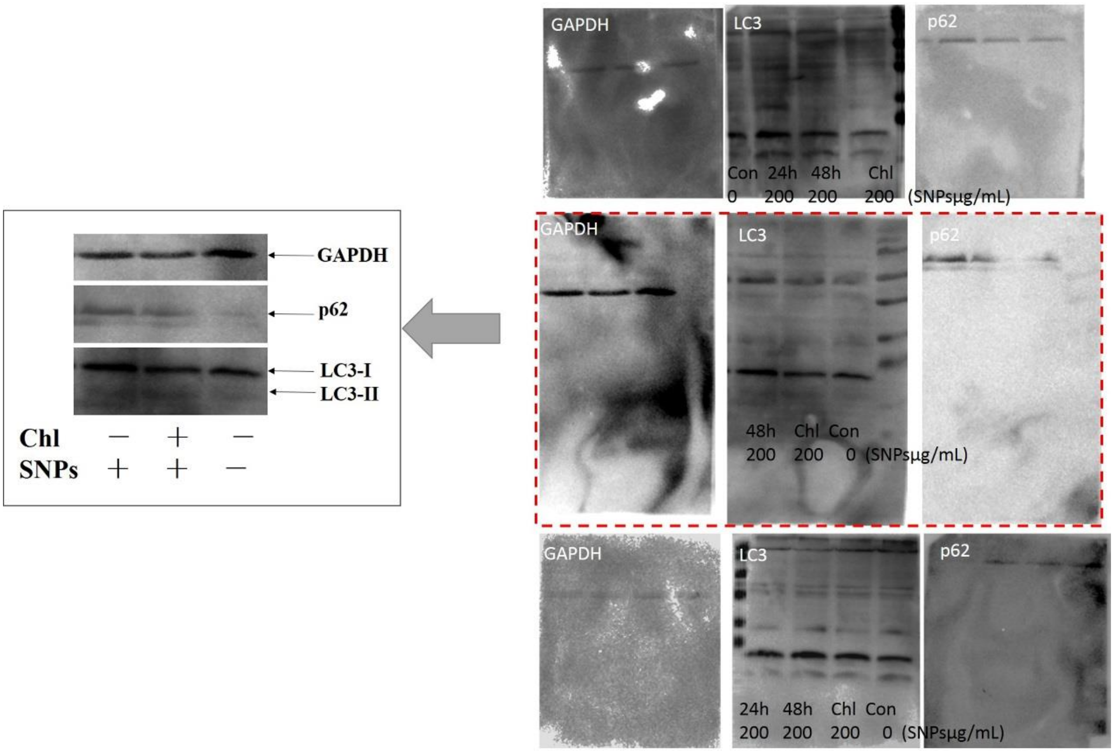

Supplementary Figure 5. SNPs induce cell autophagy. All the blotting bands were

obtained under the same experimental condition.
